# Reduction Behavior and Melting Characteristics of Blast Furnace Iron Ore Mixed with Carbon-Rich Iron Particles

**DOI:** 10.3390/ma19020248

**Published:** 2026-01-08

**Authors:** Jyun-Ming Shen, Chi-Ming Lin, You-Ren Hong, Shao-Feng Luo, Yu-Yang Chen, Jia-Shyan Shiau, Weite Wu

**Affiliations:** 1 Department of Materials Science and Engineering, National Chung Hsing University, Taichung City 402202, Taiwan; 2 Academy of Circular Economy, National Chung Hsing University, Taichung City 402202, Taiwan; linchiming5724@dragon.nchu.edu.tw (C.-M.L.);; 3 China Steel Corporation, Kaohsiung City 812010, Taiwan

**Keywords:** hot briquetted iron, blast furnace, mixed iron ores, high temperature reduction

## Abstract

The currently available hot briquetted iron (HBI) typically contains approximately 1 wt.% carbon. In the CO–CO_2_ atmosphere of a blast furnace, carbon loss from iron is significant, accompanied by overoxidation. Based on the high metallicity of HBI, this study designed iron particles with varying carbon contents. These pellets were mixed with three typical blast furnace iron ores–sinter, pellet, and lump– and subjected to thermogravimetric analysis reduction experiments. The investigation explored the effects of substituting 15 wt.% sinter with HBI containing different carbon contents and assessed the resulting impact on the temperature difference between iron and slag melting, ultimately determining the optimal carbon content for blast furnace operations. The findings showed that the addition of iron particles with carbon contents exceeding 1.6 wt.% achieved reduction rates and iron–slag melting characteristics similar to those of typical blast furnace charges. When iron particles containing 3.6 wt.% carbon were added, the iron oxides of various valence states in the charge and pellets exhibited the highest availability of carbon for both direct and indirect reduction. Consequently, the slag melting temperature rose to 1398 °C. Due to the presence of unreacted carbon, the molten iron melted at approximately 1530 °C, while the iron–slag dripping temperature range narrowed to 132 °C, achieving the optimal temperature range for blast furnace application.

## 1. Introduction

The blast furnace ironmaking process is an important technology for iron production in the world [1]. In this process, layers of iron ore and fossil fuels are alternately stacked. On the one hand, coke directly reduces the iron ore; on the other hand, the combustion of coke generates CO gas, which indirectly reduces the iron ore. The Fe_2_O_3_ present in the ore is gradually reduced to metallic Fe [2,3,4]. Therefore, the gas properties, reduction degree, and softening–melting characteristics of the ore play a vital role in determining the efficiency of the blast furnace process. Recent studies have extensively examined the effect of different oxides (CaO, SiO_2_, Al_2_O_3_, MgO, etc.) on the reduction degree and softening behavior of both single [5,6,7] and mixed [8,9,10] ironmaking raw materials, such as sinter, pellet, and lump ore. For instance, Li et al. [8] reported that an increase in the proportion of lump ore resulted in a decrease in CaO content in the primary slag, accompanied by an increase in FeO and SiO_2_ levels. This compositional change lowered the melting temperature of the primary slag and deteriorated gas permeability. Ma et al. [10] proposed that increasing the MgO content in pellets promoted the formation of high-melting-point solid phases in the slag system, thereby increasing viscosity, reducing fluidity, and worsening the permeability of the cohesive zone. Similarly, Li et al. [11] observed that, in a mixed burden, the softening properties improved, whereas the melting properties worsened with increasing MgO/Fe_2_O_3_ ratio. Considering the reduction behavior of iron ore and the thermal state of the blast furnace hearth, the optimal softening–melting properties of the mixed burden were achieved at an MgO/Al_2_O_3_ ratio of 0.82.

In response to the global trend of carbon reduction [12,13,14], many industries face the urgent challenge of effectively lowering carbon emissions. Among these, the steel industry holds particular significance, accounting for approximately 7% of total anthropogenic CO_2_ emissions [15]. Within this sector, the blast furnace ironmaking process is the primary source, accounting for more than 70% of the CO emissions generated during ironmaking [16,17]. Sintered ore constitutes nearly 70% of the raw materials used in blast furnace iron production, and its production releases pollutants such as sulfur dioxide (SO_2_), nitrogen oxides (NOx), and CO_2_, refs. [18,19,20] resulting in frontend raw material carbon emissions [21]. Furthermore, in the blast furnace process, molten iron is produced by reducing iron oxides of various valence states in iron ores using coke and a CO-containing reducing atmosphere, producing a large amount of CO_2_ emissions [22,23]. To mitigate carbon emissions from the blast furnace process, strategies can be implemented from both the process and raw material perspectives.

In terms of ore selection, low-carbon ironmaking raw materials reduced by hydrogen are widely promoted in the market. One such material is direct reduced iron (DRI), which is typically produced in a vertical furnace [24,25,26]. The DRI is then hot pressed to form high-density hot-briquetted iron (HBI), facilitating easier transportation [27]. HBI contains 80–90 wt.% metallic Fe with minimal iron oxide content. Therefore, incorporating DRI or HBI into the blast furnace as a replacement for sinter can reduce the demand for high-carbon sintered materials and decrease the reduction energy required in the blast furnace. Consequently, this approach can significantly reduce CO_2_ production and overall carbon emissions from the blast furnace [27,28].

However, achieving a high reduction degree and ensuring optimal softening and melting properties of both slag and molten iron are crucial for stable furnace operation. Correspondingly, the slag should exhibit appropriate basicity [7,29], while the molten iron should maintain a sufficient carbon content. A large disparity in melting properties can adversely affect the permeability of the blast furnace charge and compromise operational stability. Establishing component modulation technology based on HBI characteristics represents a major research direction deserving of in-depth investigation. Therefore, the main objective of this study is to conduct experiments using iron-based raw materials with varying carbon contents to simulate the high metallic iron characteristics of HBI. Iron particles with different carbon contents were designed and mixed with iron ores to simulate blast furnace reduction experiments. The study aimed to explore the influence of carbon content in HBI on the melting behavior of the overall charge and to study the optimal carbon content parameters of HBI for blast furnace applications. This approach ensures that, when introduced into the blast furnace, the overall ore reaction proceeds smoothly, the desirable melting characteristics of the iron−slag are presented, and good permeability is maintained.

## 2. Materials and Methods

### 2.1. Material Preparation and Experimental Ore Proportion

Three kinds of iron particles with varying carbon contents were prepared. Steel blocks were loaded in graphite crucibles and smelted using an induction furnace (Jia Shing Electricity Construction Co., Kaohsiung, Taiwan). Samples were collected at different time intervals using a vacuum glass rod for carbon content analysis. The carbon content of each sample was determined using a carbon and sulfur analyzer (HORIBA, Kyoto, Japan; EMIA-920). Iron ingots with different carbon contents of 1.6 wt.%, 2.9 wt.%, and 3.6 wt.% were produced and subsequently cut into particles with a diameter of 10 mm, completing the preparation of iron particles with different carbon contents.

The experiment used three iron ores–sinter, pellet, and lump ore–along with HBI-1, all obtained from domestic steel companies. The oxide contents were analyzed by WDXRF, while iron oxides with different valence states were measured using chemical wet titration. Their chemical compositions are shown in Table 1 and Table 2. To ensure the smooth operation of the blast furnace, the CaO/SiO_2_ ratio of the final slag was maintained at 1.0–1.1. However, an excessive substitution of sintered ore with iron particles could lower the basicity, thereby affecting the composition of the final slag in the blast furnace. According to Huitu et al. [28], the blast furnace process was optimized by adding 10 wt.% HBI. According to the study by Yilmaz et al. [30], partial replacement of iron-bearing raw materials in the blast furnace with DRI can reduce fuel consumption and improve productivity. In addition, studies by Chen et al. [16] and Ma et al. [31] reported that a 1% increase in the iron grade of the blast furnace burden resulted in a 1.5% decrease in fuel ratio and a 2.5% increase in pig iron production. Building upon this, the present study conducted a forward-looking experiment from an academic perspective. At a slightly higher limit than the current on-site HBI input in blast furnaces, four types of carbon-containing iron particles were used to replace sinter at a proportion of 15 wt.%. The iron particles were mixed with three types of iron ore materials—sinter, pellet, and lump ore—to conduct reduction experiments. The groups were denoted as C0.6, C1.6, C2.9, and C3.6. Taking C0.6 as an example, it represents the replacement of sinter with iron particles containing 0.6 wt.% carbon. The control group consisted of the three iron ore materials—sinter, pellet, and lump ore—and was denoted as SPL. In total, five parameter sets were employed for the 150 g high-temperature reduction experiments, as summarized in Table 3.

### 2.2. Reduction Experiment

#### 2.2.1. Experimental Apparatus for Thermogravimetric Analysis

**Figure 1:**
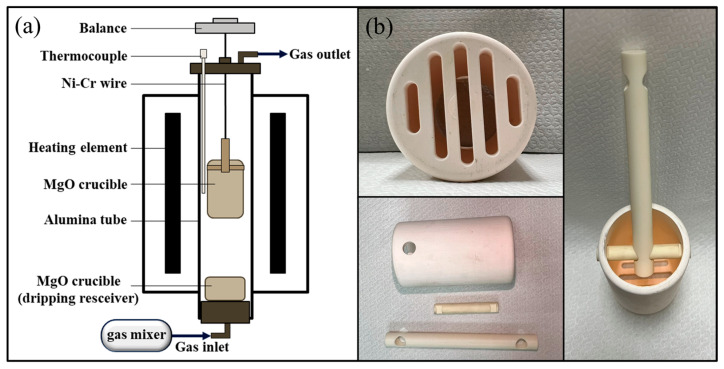
Schematic diagram of the experimental setup (**a**) TGA equipment (**b**) MgO crucible design.

**Figure 2:**
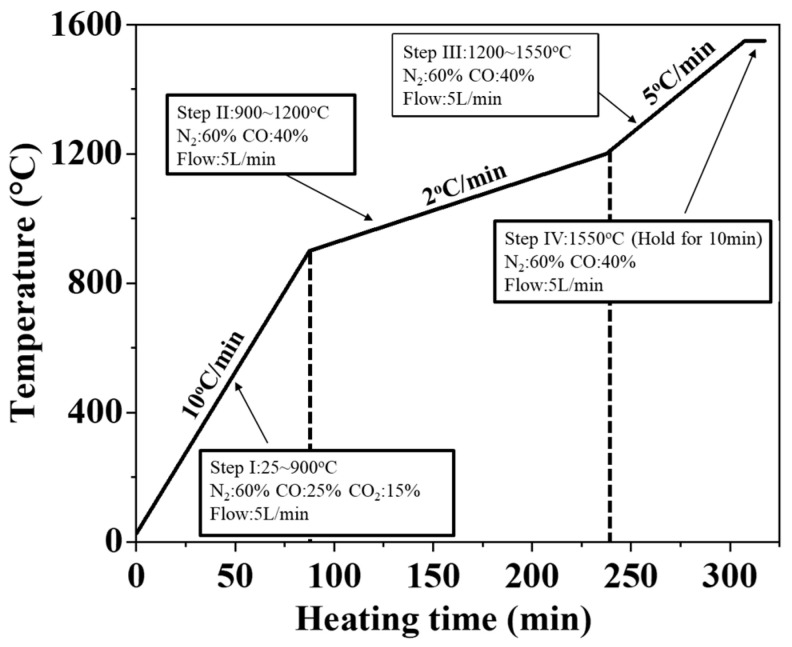
Heating rate and reducing atmosphere in the TGA experiment.

In this study, a self-built thermogravimetric analyzer (Figure 1a) was utilized, incorporating a microbalance mounted on a vertical tubular high-temperature furnace. A magnesium oxide crucible containing the furnace charge was suspended using a nickel-chromium wire. To prevent the wire from breaking at elevated temperatures, an alumina rod was connected to the crucible at the furnace center, as shown in Figure 1b. The experimental atmosphere and the temperature profile are shown in Figure 2. To simulate the blast furnace environment. The CO–CO_2_–N_2_ mixed gas was used to simulate the atmosphere inside a blast furnace. The study refers to the work of Zhang et al. [32] and Mizogu et al. [33]. The heating rates in the step I (20–900 °C), step II (900–1200 °C), and step III (1200–1550 °C) regions were 10 °C/min, 2 °C/min, and 5 °C/min, respectively. The experimental atmospheres for each stage were as follows: Step I (60%N_2_–15%CO_2_–25%CO), Step II (60%N_2_–40%CO), Step III (60%N_2_–40%CO), and Step IV (60%N_2_–40%CO). The total gas flow rate was fixed at 5 L/min.

Degree of Reduction (%R) = (weight of O_2_ removed during  process/theoretical weight of removeable O_2_ in Fe_2_O_3_ and FeO of iron ore) ×  100%,(1)

Two types of signals can be obtained from the weight loss recorded by the balance: the reduction degree of the iron ore and its melting behavior. In terms of reduction degree, the ratio of the theoretical total oxygen weight to the lost oxygen weight for the reduction in various valence iron oxides to metallic iron was calculated (Formula (1)) [34] based on the chemical composition of three typical blast furnace raw materials (Table 2). This ratio provides the reduction degree of the furnace charge throughout the process. In terms of melting behavior, when the slag and molten iron reach their melting points, the system transitions from the solid phase to the solid–liquid coexistence zone, eventually becoming fully molten and dripping, resulting in a drastic weight loss. The thermogravimetric analysis (TGA) curves distinctly show the melting differences between slag and molten iron under different experimental parameters. A collecting tray was set under the furnace tube to gather the molten products. The slag composition was first analyzed using wavelength-dispersive X-ray fluorescence spectroscopy (WDXRF, Rigaku Supermini 200, Rigaku, Tokyo, Japan), followed by viscosity measurements to assess its permeability.

#### 2.2.2. Iron Ore Stacking Method for Reduction Experiment

**Figure 3:**
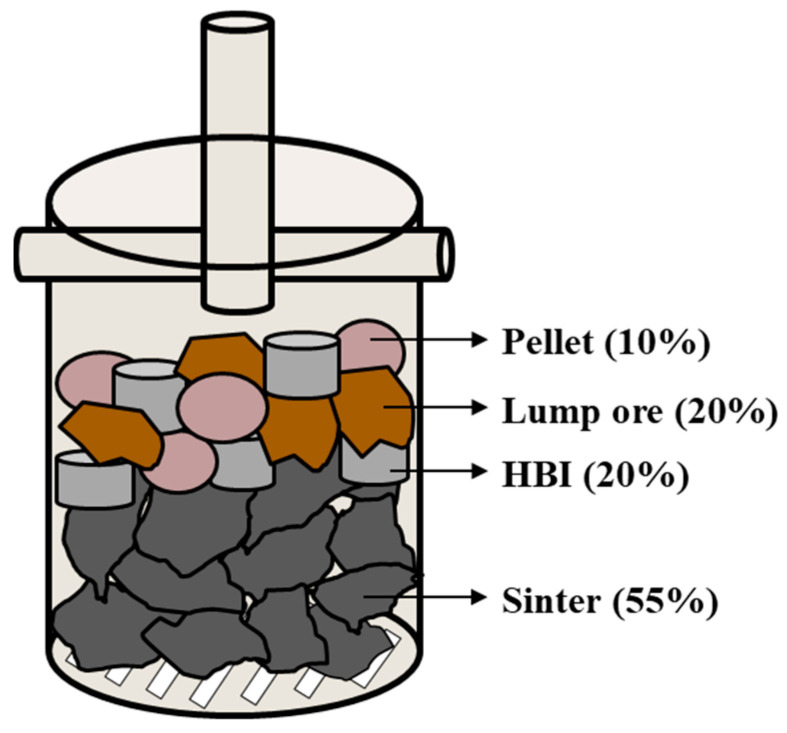
Schematic Diagram of Iron Ore Stockpiling.

Iron ore stacking represents a crucial aspect of the experimental setup. In this study, a microbalance was employed to integrate the signals. As a natural mineral material, lump ore has a low basicity (CaO/SiO_2_), which makes it prone to reduction-induced swelling and disintegration. To minimize experimental errors caused by iron ore crushing [29]. Therefore, the sintered ore was placed at the bottom of the crucible to support the other iron ores, ensuring minimal data distortion caused by high-temperature expansion during the reaction process. Carbon-containing iron particles, pellet, and lump ore were then dispersedly stacked on top of the sinter. This stacking method allowed carbon-containing iron particles to simultaneously contact the three iron ores, thereby facilitating exploration of the reduction pathways and melting behaviors of carbon elements in the mixed ore under varying carbon content parameters, as illustrated in Figure 3.

### 2.3. Primary Slag Viscosity Analysis

#### 2.3.1. Sample Preparation for Viscosity Analysis

To investigate the fluidity of slag within the blast furnace after replacing sinter with HBI, viscometry was employed to measure slag viscosity. The composition of the primary slag obtained from the TGA mixed iron ore reduction test was first determined using WDXRF (Table 4). Viscosity test samples were prepared from reagent-grade powders of CaO, SiO_2_, Al_2_O_3_, MgO, and FeO. To ensure uniform mixing, the powders were thoroughly mixed and stirred at room temperature. The uniformly mixed powder sample was then placed in a graphite crucible. The sample was heated to 1600 °C in a high-frequency induction furnace (FTR-25H, Five Powers Electric Machinery, Taipei, Taiwan). Once the powders were completely melted into a liquid state, the melt was rapidly cooled in water to achieve an appropriate temperature, completing the preparation of the viscosity test samples.

#### 2.3.2. Procedure of Viscosity Measurements

**Figure 4:**
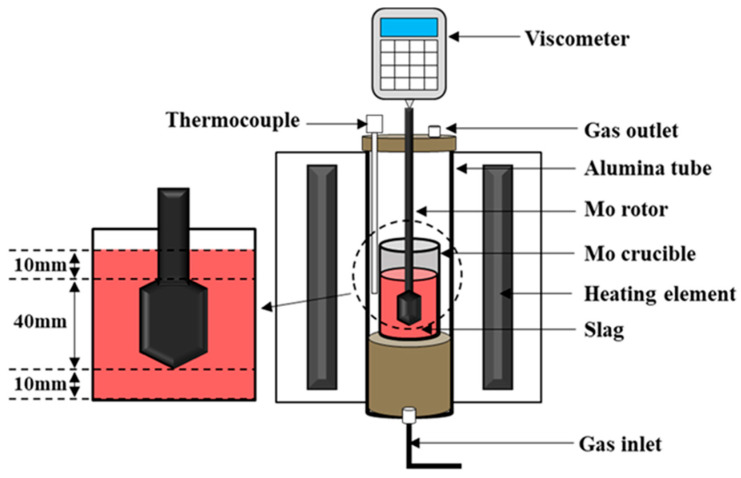
Schematic diagram of the viscosity experimental device.

Slag viscosity was measured using a viscometer (Brookfield DV IIIRV, AMETEK Brookfield, Middleboro, MA, USA) in conjunction with a vertical tubular high-temperature furnace (SJ High Technology Company, Taipei, Taiwan). The schematic of the equipment diagram is shown in Figure 4. A premelted slag sample weighing 140 g was placed in a molybdenum crucible and heated in the tubular furnace. The temperature was monitored using a B-type thermocouple. The heating rate was maintained at 10 °C/min from 0 °C to 1300 °C and 2 °C/min from 1300 °C to 1600 °C. Nitrogen gas was supplied at a constant flow rate of 8 L/min as a shielding gas throughout the measurement. After the slag was partially liquefied, a molybdenum rotor was slowly lowered to a position 10 mm above the crucible bottom. The viscometer speed was set to 20 rpm, and viscosity measurements were initiated. Data collection continued until the temperature reached 1600 °C, after which the torque values were converted to viscosity values (Pa·s) using the appropriate calibration formula.

## 3. Results

### 3.1. High-Temperature Reduction and Melting Behavior

**Figure 5:**
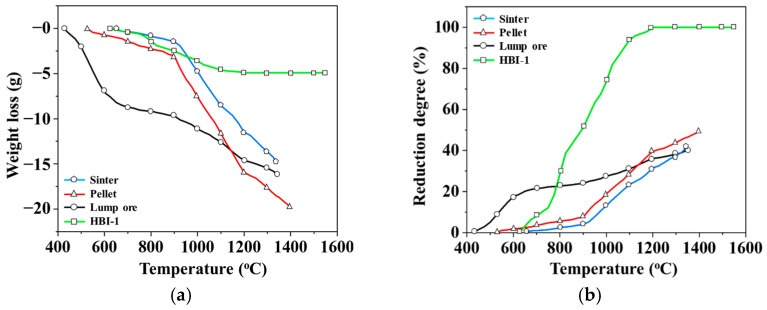
TGA reduction behavior of an iron ore: (**a**) weight loss; (**b**) reduction degree.

**Figure 6:**
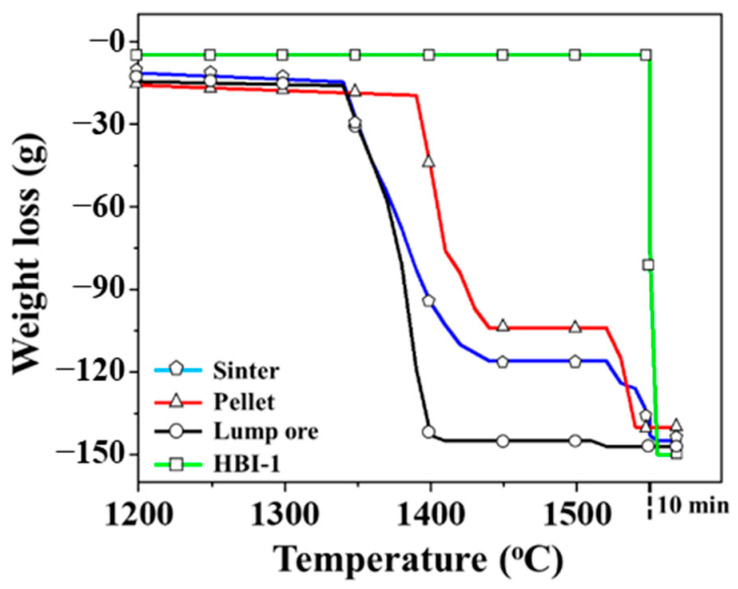
Weight loss in an iron ore reduction experiment.

In order to understand the reduction characteristics of an iron ore, a high-temperature TGA experiment was conducted using 150 g samples of three blast furnace iron ores (sinter, pellet, and lump ore) and HBI-1. Figure 5a shows the weight loss profiles of the four materials during the reaction process. The reducing gas used was a mixture of CO and CO_2_. According to the chemical reactivity of the elements, the observed weight change is attributed to the release of oxygen from iron oxides with various valence states. Based on this, the total oxygen content in these oxides was theoretically calculated based on the reduction degree (Equation (1)) to understand the differences in reduction degree of different materials. Figure 5b shows the variation in reduction characteristics of the four materials before slag melting. The lump ore exhibits a violent reduction reaction around 425 °C, which gradually slows down as the temperature exceeds 700 °C. In contrast, the reduction degree trends of sinter and pellet exhibit a similar pattern, with both showing a significant increase beyond 900 °C. Based on the combined analysis of these characteristics, lump ore, as a natural mineral material, has a low basicity (CaO/SiO_2_) and is therefore prone to reduction swelling. During the reduction of Fe_2_O_3_ to Fe_3_O_4_, a crystal structure transformation occurs [29,35], changing from a trigonal crystal system to an isoaxial crystal system. Due to the differences in crystal structure, this transformation leads to significant volume expansion. Subsequently, during further reduction to FeO and Fe, the loss of O^2–^ ions causes volume contraction, accompanied by the formation of numerous internal pores and cracks. In addition, lump ore contains crystallization water [36], which is released as the temperature increases. The diffusion of part of the water vapor is hindered by closed pores, resulting in the accumulation of local vapor pressure inside the lump ore. Eventually, bursting initiates and propagates along structurally weak regions. Consequently, lump ore is susceptible to reduction swelling, fragmentation, and disintegration during the reduction process. Upon reaching the melting point, these fragmented fine lump ore particles melt rapidly and drip downward together with the reduced fine metallic iron particles. As observed in Figure 6, the melting–dripping signal of lump ore is steeper than that of the other materials, and no distinct dripping signal of molten metallic iron was detected. This leads to increased contact area with the reducing gas, thereby enhancing its reducing ability in the low-temperature zone. When the temperature reaches approximately 1150 °C, the reduction rate of the pellet becomes slightly higher than that of the sinter and lump ore. Moreover, its slag remains unmelted until approximately 1400 °C, resulting in a final reduction rate approximately 10% higher than that of the other two materials. The iron ore reduction test indicates that the addition of pellets and sinter not only provides the maximum yield of reduced iron but also increases the melting temperature of the overall slag. In the HBI-1 group, since the HBI material contains only a very small amount of FeO, the required reduction energy is relatively lower, and a complete (100%) reduction is achieved at approximately 1200 °C. This confirms the carbon reduction strategy of incorporating HBI into blast furnaces as a partial substitute for sinter in the future.

Figure 6 presents the TGA curve of an iron ore sample in the high-temperature zone (>1200 °C). The differences in melting behavior among the groups are distinguished based on the sharp weight drop signals. For the three common iron ores used in blast furnaces, the weight loss trends of sinter and pellet are nearly identical, each exhibiting two distinct weight loss signals. From the melting temperature ranges of slag and metallic iron, these stages can be attributed to the sequential slag melting, followed by the reduction and iron melting. Moreover, the slag in the pellet exhibits a notably higher melting temperature than that in the sinter, and the melting range of iron and slag is smaller, suggesting superior melting characteristics. In contrast, the melting curve of lump ore does not exhibit the same trend. A sharp weight loss is evident at about 1340 °C, indicating that lump ore may not be reduced to metallic iron under the given reduction conditions. In the HBI-1 group, only a slight weight loss occurs, as the original HBI contains more than 80 wt.% of metallic iron. As shown in Figure 5b, it has been almost completely reduced after the reaction, with negligible slag content. Consequently, the melting of metallic iron occurs only at 1550 °C.

### 3.2. Reduction Degree of Mixed Composite Iron Ore with Different Carbon Contents

**Figure 7:**
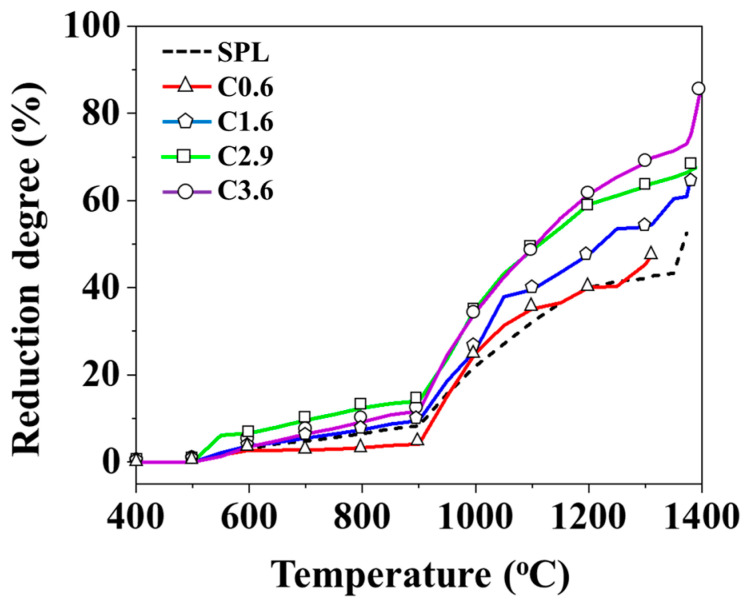
Reduction degree of mixed iron ore with different carbon contents.

Figure 7 shows the difference in the reduction degree of mixed iron ore when four different carbon-containing iron particles replace 15 wt.% of sinter. The dotted line represents the SPL (control group), which is composed of three standard blast furnace iron ores. The objective is to identify the optimal HBI carbon content parameter for sinter replacement under a fixed substitution ratio based on the reduction degree. The reduction curves indicate that the differences in reduction degree remain minimal below 900 °C. At lower temperatures and shorter reaction times, the carbon content in the iron has little influence on the reduction behavior of the three typical furnace charges. However, when the temperature exceeds 900 °C, the reduction degrees of C1.6, C2.9, and C3.6 become significantly higher than those of the SPL. Before the slag melts, the final reduction degrees are 66%, 68%, and 86%, respectively, while that of the SPL is 53%.

**Figure 8:**
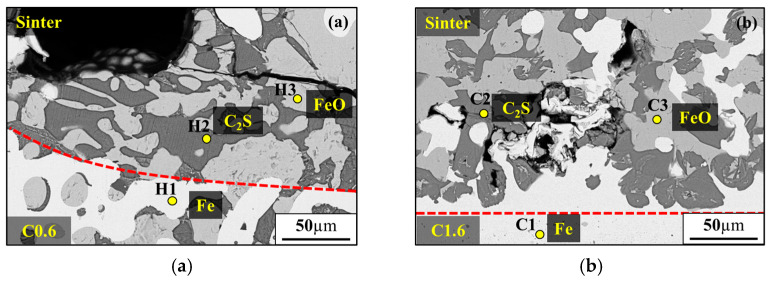
SEM observation of iron particles and sinter interface: (**a**) C0.6; (**b**) C1.6.

When the carbon content in the iron particles surpasses 1.6 wt.%, the reduction degree is comparable to that of the traditional blast furnace iron ore batch composition, and increases further with rising carbon content. The interfacial reaction between iron particles and the sinter at 1200 °C was observed by SEM-EDS (Figure 8 and Table 5). The red dashed line indicates the interface between the iron particles and the sinter. It was observed that, for iron particles containing 1.6 wt.% carbon, a higher amount of metallic iron was present above the dashed line (Figure 8b). This can be attributed to the carbon in the iron particles, which promotes the reduction in the surrounding iron ore. This result further confirms that an iron particle carbon content higher than 1.6 wt.% can enhance the reduction degree. In contrast, the C0.6 group exhibits a slightly lower final reduction degree than that of the SPL. This phenomenon is likely ascribed to the insufficient carbon content in the pellets. Under a reducing atmosphere, the stable phases of CO and CO_2_ vary with temperature, and the iron particles lack sufficient carbon to protect the iron substrate. Therefore, metallic iron reacts with CO gas to form peroxide [34], resulting in a final reduction degree of around 48%, the lowest among all groups.

### 3.3. Melting Behavior of Mixed Iron Ore with Different Carbon Contents

**Figure 9:**
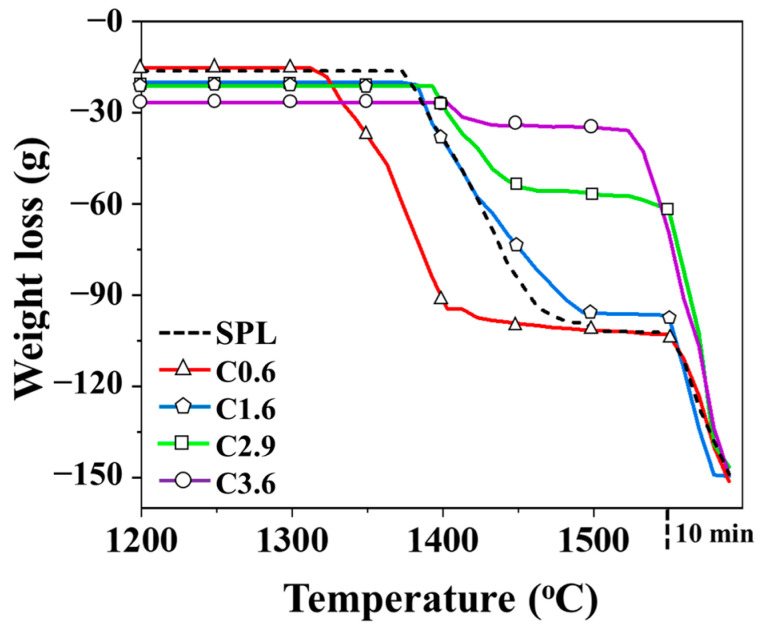
Weight loss behavior of mixed iron ore containing different carbon contents.

**Figure 10:**
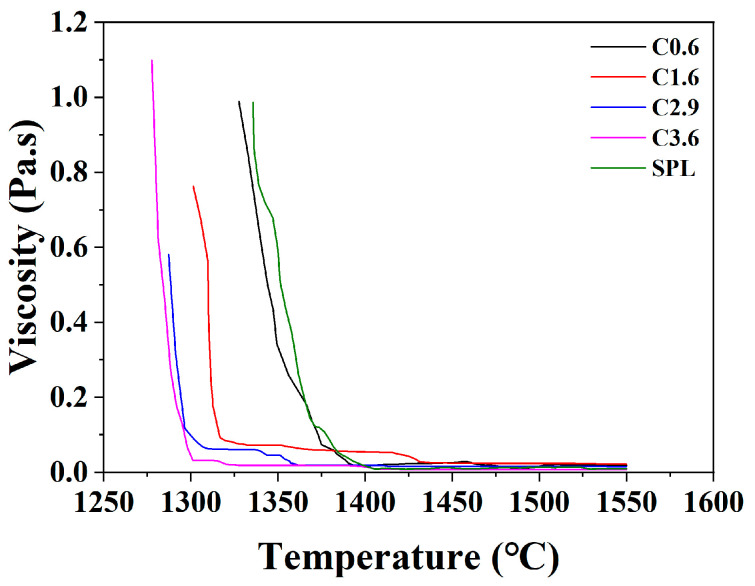
Analysis of slag viscosity in the TGA reduction test of mixed ores.

Figure 9 depicts the variation in weight loss of the whole mixed iron ore when four types of iron particles with different carbon contents are used to replace 15 wt.% sinter. The dotted line denotes the SPL (control group). Based on the TGA curve, the slag melting temperature in the SPL is 1373 °C, the molten iron melting temperature is 1550 °C, and the iron–slag melting range is roughly 177 °C. When iron particles containing 0.6 wt.% carbon are added to replace 15 wt.% sintered ore, the slag melting temperature decreases rather than increases, causing an expansion of the iron–slag melting range to 238 °C. The corresponding interval time is about 12 min longer than that of the SPL, which is not conducive to blast furnace operation. As the carbon content of the iron particles increases to 1.6 wt.%, 2.9 wt.%, and 3.6 wt.%, the slag melting temperature rises to 1381 °C, 1389 °C, and 1398 °C, respectively. It can be noted that, with an increase in carbon content, the reduction rate increases, while the FeO content in the slag decreases. The weaker heterogeneous bonds formed among the CaO-SiO_2_-Al_2_O_3_-MgO-FeO polyoxides diminish, causing the mixed slag sample to require a higher temperature to break these bonds and achieve melting. According to the viscosity analysis results (Figure 10), the viscosity values remain below 0.6 Pa·s above 1400 °C. As reported by Wang et al. [8], the viscosity of slag reflects the friction coefficient between different flow layers. Higher viscosity indicates poorer fluidity, which can lead to clogging and reduced permeability in the blast furnace. Nakamoto et al. [35] further reported that a slag viscosity below 0.6 Pa·s ensures sufficient fluidity during blast furnace operation at 1673 K. Analysis of the carbon content in the dripping metallic iron after reduction (Table 4) revealed that the carbon content of iron in group C3.6 is slightly higher than that of the other three groups by 0.03 wt.%. This group also showed the highest total amount of reduced iron, resulting in earlier molten iron dripping at 1530 °C. Consequently, the melting and dripping temperature range between iron and slag is narrowed to 132 °C, helping to prevent issues such as blockages caused by large differences in dripping properties.

### 3.4. Iron–Slag Melting Temperature of Mixed Ore Reduction Test

**Figure 11:**
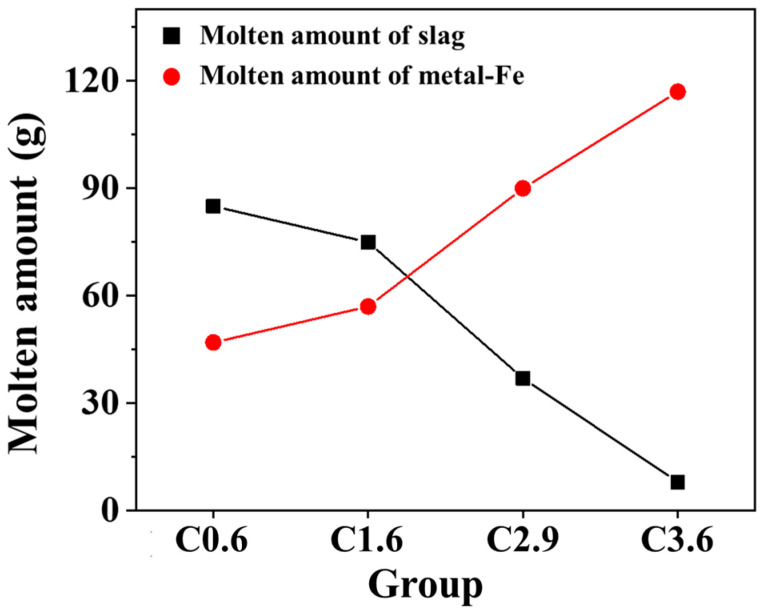
Total amount of iron–slag melt of mixed iron ore with different carbon content.

The TGA analysis revealed that the extent of iron–slag melting in the mixed ore varies significantly with the addition of iron particles containing different carbon contents (Figure 11). As the carbon content of the iron particles increases, the total amount of molten iron increases, while the amount of slag dripping decreases. These findings indicate that carbon in iron effectively acts as a reducing agent.

**Figure 12:**
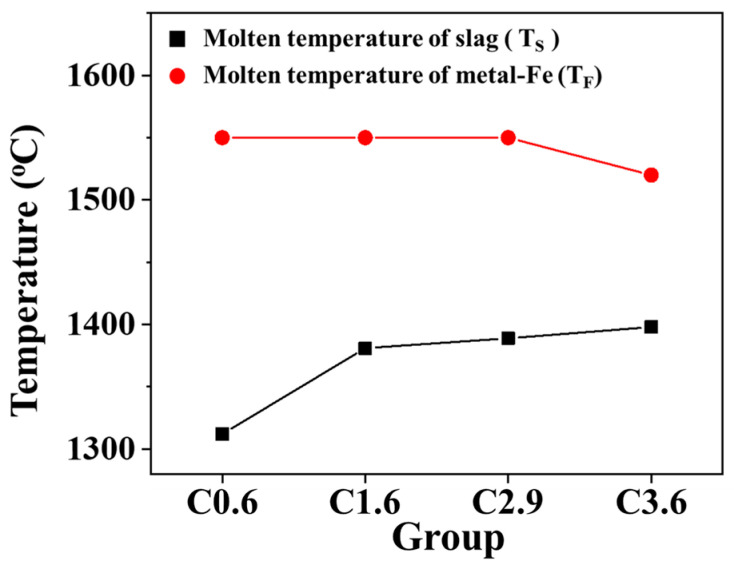
Melting temperature range of iron and slag in mixed iron ore containing iron particles with different carbon contents.

In blast furnace operations, the temperature ranges of molten iron (T_F_) and molten slag (T_S_) are critical parameters that directly affect the stable performance of the blast furnace. A shorter sequential melting time of these two substances indicates a reduced risk of material blockage in the lower part of the furnace. Figure 12 presents the melting ranges of iron and slag for mixed ores containing iron particles with varying carbon contents. As the carbon content in the iron particles increases from 0.6 wt.% to 3.6 wt.%, the combined melting temperature range of iron and slag decreases from 238 °C to 122 °C, corresponding to a reduction in melting duration of approximately 23 min. It is evident that the addition of carbon to iron directly affects the melting temperature of slag, while simultaneously reducing the melting temperature range of both iron and slag, which is beneficial to the operation of the blast furnace.

Based on the above studies, considering both cost and carbon emissions, substituting sinter with HBI to enhance productivity can achieve the intended objective when the carbon content of HBI reaches 1.6 wt.%. When 15 wt.% HBI is used to replace sinter (Formulas (2) and (3)) [17], the iron grade of the blast furnace burden increases from 59.19 wt.% to 64.28 wt.%, the fuel ratio decreases by 7.64%, and pig iron production increases by 12.73%, thereby achieving the goals of carbon emission reduction and increased productivity.

(2) Decrease in fuel ratio (%)=∆TFe × 1.5%

(3) Increase in pig iron production (%)=ΔTFe × 2.5%

## 4. Conclusions

This study used TGA to simulate the temperature profile and reduce the atmosphere of an actual blast furnace. Blending experiments were conducted using typical blast furnace iron ore and various carbon-rich, high-metallic materials. The optimal carbon content of HBI for efficient blast furnace operation was determined based on key operational criteria. The results from multiple blends of high-metallic materials with varying carbon contents revealed that higher carbon contents not only enhance reduction kinetics but also shorten the iron–slag melting and dripping interval, thereby maintaining superior iron–slag melting characteristics and stable blast furnace operation. From these observations, the following conclusions are drawn:

1. When the carbon content of the high-metallic material was 0.6 wt.%, it exhibited the lowest reduction rate (47%) and the widest iron–slag dripping temperature range (238 °C). When the carbon content exceeded 1.6 wt.%, both the reduction degree and the iron–slag dripping temperature range were superior to those of conventional blast furnace iron ore blends.
2. Increasing the carbon content of the high-metallic material from 1.6 wt.% to 3.6 wt.% increased the reduction rate from 65% to 86%, representing a 21% improvement. At a carbon content of 3.6 wt.%, the iron–slag dripping temperature range decreased to 122 °C, achieving an optimal temperature range.
3. Theoretical prediction indicated that replacing 15 wt.% of sinter with HBI containing 1.6 wt.% carbon could yield comparable reduction rates and slag melting characteristics to those of traditional blast furnace materials. Therefore, 1.6 wt.% is identified as the optimal carbon content in HBI for adjusting blast furnace slag.

## Figures and Tables

**Table 1:** Chemical compositions of three commercially available HBI-1.

Sample	Chemical Composition, wt.%								
	Fe	C	FeO	Fe_2_O_3_	CaO	SiO_2_	C/S	Al_2_O_3_	MgO
HBI-1	79.97	0.64	13.67	0.71	1.14	2.57	0.44	0.99	0.15

**Table 2:** Chemical composition of iron ore samples, wt.%.

Sample	Chemical Composition, wt.%					
	CaO	SiO_2_	Al_2_O_3_	MgO	FeO	Fe_2_O_3_
Sinter	9.59	5.08	1.76	1.33	5.43	76.30
Pellet	2.13	2.93	0.67	0.18	0.26	93.61
Lump	0.09	2.49	1.11	0.01	0.53	89.48

**Table 3:** Proportions of constituent raw materials in the charging mixes, %.

Group	Sinter	Pellet	Lump Ore	Iron Particles
SPL	70	10	20	-
C0.6	55	10	20	15 (0.6 wt.% C)
C1.6	55	10	20	15 (1.6 wt.% C)
C2.9	55	10	20	15 (2.9 wt.% C)
C3.6	55	10	20	15 (3.5 wt.% C)

**Table 4:** WDXRF Analysis of Slag Composition from Reduction Experiments.

Sample	Chemical Composition, wt.%				
	CaO	SiO_2_	Al_2_O_3_	MgO	FeO
SPL	10.9	6.3	11.5	2.4	68.9
C0.6	12.9	10.7	5.6	3.1	67.7
C1.6	16.8	13.9	3.7	3.3	62.3
C2.9	22.5	18.6	2.7	2.9	53.3
C3.6	26.6	22.2	2.3	3.4	45.5

**Table 5:** EDS composition analysis of iron particles and sinter interface.

No.	Chemical Composition, at.%						Phase
	Fe	C	Ca	Si	Mg	O	
H1	84.3	15.7	-	-	-	-	Fe
H2	0.6	-	27.3	14.1	-	58	C_2_S
H3	53.2	-	1.2	-	3.7	41.9	FeO
C1	76.3	23.7	-	-	-	-	Fe
C2	0.7	-	29.3	14.5	-	55.5	C_2_S
C3	52.8	-	-	-	-	47.2	FeO

## Data Availability

The original contributions presented in this study are included in the article. Further inquiries can be directed to the corresponding author.
